# Identification of Genomic Predictors of Muscle Fiber Size

**DOI:** 10.3390/cells13141212

**Published:** 2024-07-18

**Authors:** João Paulo L. F. Guilherme, Ekaterina A. Semenova, Naoki Kikuchi, Hiroki Homma, Ayumu Kozuma, Mika Saito, Hirofumi Zempo, Shingo Matsumoto, Naoyuki Kobatake, Koichi Nakazato, Takanobu Okamoto, George John, Rinat A. Yusupov, Andrey K. Larin, Nikolay A. Kulemin, Ilnaz M. Gazizov, Edward V. Generozov, Ildus I. Ahmetov

**Affiliations:** 1 Human Genome and Stem Cell Research Center, University of São Paulo, São Paulo 05508-090, SP, Brazil; 2 Department of Molecular Biology and Genetics, Lopukhin Federal Research and Clinical Center of Physical-Chemical Medicine of Federal Medical Biological Agency, 119435 Moscow, Russiagenerozov@gmail.com (E.V.G.); 3 Research Institute of Physical Culture and Sport, Volga Region State University of Physical Culture, Sport and Tourism, 420138 Kazan, Russia; 4 Graduate School of Health and Sport Science, Nippon Sport Science University, Tokyo 158-8581, Japanmatsumoto-s@nittai.ac.jp (S.M.); nakazato@nittai.ac.jp (K.N.);; 5 Graduate School of Health and Sports Science, Juntendo University, Chiba 270-1695, Japan; 6 Faculty of Health and Nutrition, Tokyo Seiei College, Tokyo 124-8530, Japan; 7 Transform Specialist Medical Centre, Dubai 119190, United Arab Emirates; 8 Department of Physical Culture and Sport, Kazan National Research Technical University Named after A.N. Tupolev-KAI, 420111 Kazan, Russia; 9 Department of Human Anatomy, Kazan State Medical University, 420012 Kazan, Russia; ilnazaziz@mail.ru; 10 Laboratory of Genetics of Aging and Longevity, Kazan State Medical University, 420012 Kazan, Russia; 11 Sports Genetics Laboratory, St. Petersburg Research Institute of Physical Culture, 191040 St. Petersburg, Russia; 12 Research Institute for Sport and Exercise Sciences, Liverpool John Moores University, Liverpool L3 5AF, UK

**Keywords:** athletes, athletic status, genetic diversity, genetic predisposition, genotype, human genetics, muscle hypertrophy, muscle strength, myofibers, sports

## Abstract

The greater muscle fiber cross-sectional area (CSA) is associated with greater skeletal muscle mass and strength, whereas muscle fiber atrophy is considered a major feature of sarcopenia. Muscle fiber size is a polygenic trait influenced by both environmental and genetic factors. However, the genetic variants underlying inter-individual differences in muscle fiber size remain largely unknown. The aim of our study was to determine whether 1535 genetic variants previously identified in a genome-wide association study of appendicular lean mass are associated with the CSA of fast-twitch muscle fibers (which better predict muscle strength) in the m. vastus lateralis of 148 physically active individuals (19 power-trained and 28 endurance-trained females, age 28.0 ± 1.1; 28 power-trained and 73 endurance-trained males, age 31.1 ± 0.8). Fifty-seven single-nucleotide polymorphisms (SNPs) were identified as having an association with muscle fiber size (*p* < 0.05). Of these 57 SNPs, 31 variants were also associated with handgrip strength in the UK Biobank cohort (*n* = 359,729). Furthermore, using East Asian and East European athletic (*n* = 731) and non-athletic (*n* = 515) cohorts, we identified 16 SNPs associated with athlete statuses (sprinter, wrestler, strength, and speed–strength athlete) and weightlifting performance. All SNPs had the same direction of association, i.e., the lean mass-increasing allele was positively associated with the CSA of muscle fibers, handgrip strength, weightlifting performance, and power athlete status. In conclusion, we identified 57 genetic variants associated with both appendicular lean mass and fast-twitch muscle fiber size of m. vastus lateralis that may, in part, contribute to a greater predisposition to power sports.

## 1. Introduction

The human body contains >600 skeletal muscles and mainly comprises long contractile multi-nucleated cells called muscle fibers [1]. The architecture of skeletal muscle is characterized by a very particular and well-described arrangement of muscle fibers and associated connective tissue [2], which contribute to important mechanical and metabolic functions. Skeletal muscle hypertrophy characteristics can be presented on a micro-level (muscle fiber size) and macro-level (muscle size). From a mechanical point of view, the main function of skeletal muscle is to convert chemical energy into mechanical energy to generate force and power (the ability to exert maximum force in the shortest amount of time), and the size of a muscle (determined mostly by the number and size of individual muscle fibers) can be a crucial factor in its mechanical efficiency [3,4]. Muscle size (which correlates with muscle fiber size) is a well-known predictor of the ability to produce force in human populations, including healthy individuals [5] and elite athletes [6].

Muscle strength (i.e., the amount of force a muscle can produce with a single maximal effort) and muscle size are different, but potentially related, traits [7]. There were high correlations (r > 0.7) between maximal dynamic strength and muscle size variables, including the muscle cross-sectional area (CSA)—the bidimensional space of a transversal view of the muscle [8]. Of particular interest, increasing the CSA and muscle strength is of crucial importance to improve physical function in health and sports performance.

Skeletal muscle is one of the most dynamic and plastic tissues of the human body and muscle. Consistent exercise training augments skeletal muscle mass and strength [1]. Muscle size and strength appear to be related to the amount and intensity of regular exercise performed [7], particularly resistance or strength training. An effort to improve strength through training follows a sequence or progression in which the muscle CSA first increases (i.e., hypertrophy) [9]. Studies indicate that skeletal muscle hypertrophy induced by different stimuli is accompanied by an increase in the expression of genes encoding enzymes involved in anabolic pathways [10,11] and changes in glucose metabolism [12]. However, the magnitude of training-induced muscle hypertrophy is highly variable across individuals [13,14,15], as seen in the CSA of fast-twitch (type II) muscle fibers of older adults following 4 weeks of the same training protocol [16]. Evidence suggests that the baseline expression of skeletal muscle gene networks can probably partially predict hypertrophic response [17] based on a genetic predisposition.

Muscle fiber size is determined by both environmental (e.g., training, nutrition, and sleep) and genetic (heritable) factors. Animal studies have found that the total contribution of heritable factors accounts for 19–32% of the variation in muscle fiber size [18,19]. These heritable factors may include DNA sequence variants, such as single-nucleotide polymorphisms (SNPs), indels, and structural variations, which can alter gene expression and/or protein structure. Indeed, genetic variants can significantly contribute to a greater muscle fiber CSA [20,21]. In recent years, there have been some attempts to identify genetic variants associated with the CSA of muscle fibers [20,21,22,23,24,25], especially fast-twitch muscle fibers, which are more responsive to hypertrophy and better predict muscle strength [26]. These studies revealed few genetic variants, but because the muscle fiber CSA is a polygenic trait, there are still a large number of genetic markers to be discovered. It is plausible to assume that the genetic context of inter-individual variability in skeletal muscle fiber size remains limited.

Therefore, the aim of this study was to discover new genetic markers associated with the fast-twitch muscle fiber size of m. vastus lateralis. We are going to test the hypothesis that 1535 genetic variants previously reported to be associated with appendicular lean mass [27] are also associated with the CSA of fast-twitch muscle fibers in physically active individuals. Given that muscle biopsy to evaluate the CSA of muscle fibers is an invasive measure, the number of subjects usually evaluated is lower than necessary to discover genome-wide significant SNPs. So, our proposal was to use a surrogate phenotype (appendicular lean mass) with powerful genome-wide significant SNPs and check if they are also associated with the CSA of fast-twitch muscle fibers. This approach has the potential to increase the probability of identifying relevant SNPs while decreasing the probability of obtaining false-positive associations. In addition, to further strengthen our results, the findings will be supported by publicly available data (the bioinformatic part of this study) from the UK Biobank (muscular strength), the Genotype-Tissue Expression (GTEx), mouse gene knockout data (The International Mouse Phenotyping Consortium), and replications in athletic cohorts (own data).

## 2. Materials and Methods

### 2.1. Ethical Approval

This study was approved by the Ethics Committees of the Federal Research and Clinical Center of Physical-Chemical Medicine of the Federal Medical and Biological Agency of Russia (reference 2017/04) and the Nippon Sport Science University (reference 021-G01). UK Biobank has approval from the North West Multi-centre Research Ethics Committee (MREC) as a Research Tissue Bank (RTB) approval (reference 11/NW/0382). Written informed consent was obtained from each participant before the start of this study, which complied with the Declaration of Helsinki and ethical standards for sport and exercise science research.

### 2.2. Study Participants

#### 2.2.1. The Russian Cohorts

The Russian muscle biopsy study involved 148 physically active participants with different training (19 power-trained and 28 endurance-trained females, 28 power-trained and 73 endurance-trained males) backgrounds of Russian origin (101 men: mean age ± SD: 31.1 ± 0.8 years; mean height: 180.2 ± 0.6 cm; mean body mass: 80.3 ± 1.0 kg; 47 women: mean age ± SD: 28.0 ± 1.1 years; mean height: 167.2 ± 0.8 cm; mean body mass: 59.7 ± 0.8 kg). They were classified according to their training frequency as mildly active (2 training sessions per week), moderately active (3–4 training sessions per week), highly active (5–7 training sessions per week), or extremely active (2 training sessions per day). Detailed histological characteristics of these subjects can be found in the Supplementary Table S1.

The Russian case–control study involved 612 elite athletes (364 males and 248 females; age 28.1 ± 4.9 years), of whom 115 were elite sprinters (29 100–400 m runners, 38 500–1000 m speed skaters, 22 sprint cyclists, 26 50 m swimmers; 75 were highly elite sprinters), 373 were elite endurance athletes (52 rowers, 32 biathletes, 7 long-distance cyclists, 30 kayakers and canoers, 37 middle- and long-distance speed skaters, 92 cross-country skiers, 63 middle- and long-distance runners, 31 middle- and long-distance swimmers, 8 race walkers, and 21 triathletes; 256 were highly elite endurance athletes), 83 were elite strength athletes (53 weightlifters, 30 powerlifters; 48 were highly elite strength athletes), and 41 were elite speed–strength athletes (3 decathletes, 2 heptathletes, 10 throwers, 26 jumpers; 27 were highly elite speed–strength athletes). Overall, sprinters (*n* = 115), speed–strength athletes (*n* = 41), and strength athletes (*n* = 83) were classified as power athletes (*n* = 239). The athletes were Russian national team members (elite athletes: participants in international competitions; highly elite athletes: prize winners of international competitions) who had never tested positive for doping. The controls were 209 healthy and unrelated citizens of Russia without any competitive sport experience (50 females, 159 males; age 44.9 ± 4.3 years).

#### 2.2.2. The Japanese Cohorts

The Japanese case–control study involved 119 athletes (83 males and 36 females), of whom 78 were strength athletes (68 weightlifters with average age of 28.5 ± 13.8 years, 10 powerlifters with an average age of 32.0 ± 6.7 years; 27 were highly elite, 27 were elite, and 24 were sub-elite), and 41 were wrestlers (34 were highly elite, and 7 were elite wrestlers; age 29.2 ± 10.2 years). Highly elite athletes were prize winners of international competitions, elite athletes were participants in international competitions, and sub-elite athletes were participants in national (Japan) competitions. The control group consisted of 306 Japanese individuals (143 males and 163 females; age 69.2 ± 11.7 years).

#### 2.2.3. The UK Biobank Cohort

Appendicular lean mass-related SNPs associated with the CSA of fast-twitch muscle fibers were subsequently tested for associations with handgrip strength, walking pace, and physical activity traits in the UK Biobank—a prospective population-based study of >350,000 individuals (summary statistics are available from https://genetics.opentargets.org/ (accessed on 25 May 2024)), as previously described [28].

### 2.3. Assessment of the Cross-Sectional Area (CSA) of Fast-Twitch Muscle Fibers

Muscle fiber composition and the cross-sectional area (CSA) of fast-twitch muscle fibers were evaluated using immunohistochemistry, as previously described [29]. Briefly, vastus lateralis samples were obtained from the left leg using the modified Bergström needle procedure with aspiration under local anesthesia using a 2% lidocaine solution. Serial cross-sections (7 μm) were obtained from frozen samples using an ultratom (Leica Microsystems, Wetzlar, Germany). The sections were then incubated at RT in primary antibodies against slow or fast isoforms of the myosin heavy chains (M8421, 1:5000; M4276; 1:600, respectively; Sigma-Aldrich, Burlington, MA, USA) for 1 h and incubated in PBS (3 × 5 min). Afterwards, the sections were incubated at RT in secondary antibodies conjugated with FITC (F0257; 1:100; Sigma-Aldrich) for 1 h. The antibodies were removed, and the sections were washed in PBS (3 × 5 min), placed in mounting media, and covered with a cover slip. Images were captured by a fluorescent microscope (Eclipse Ti-U, Nikon, Japan) (Supplementary Figure S1). All analyzed images contained 329 ± 14 fibers. The ratio of the number of stained fibers to the total fiber number was calculated.

### 2.4. Genotyping

#### 2.4.1. Russian Study

Molecular genetic analysis was performed with DNA samples obtained from leukocytes (4 mL venous blood). DNA extraction and purification were performed using a commercial kit according to the manufacturer’s instructions (Technoclon, Moscow, Russia). Genotyping of SNPs was performed using microarray technology (Illumina, San Diego, CA, USA) with HumanOmni1-Quad and HumanOmniExpress BeadChips (Illumina, San Diego, CA, USA), as previously described [30].

#### 2.4.2. Japanese Study

DNA samples were collected from the saliva using the Oragene^®^ DNA Collection Kit (DNA Genotek, ON, Canada). DNA samples were genotyped using the Japonica Array v.2 (Toshiba Co., Tokyo, Japan) for all subjects. Genotyping and SNP quality control (QC) were carried out in accordance with recommended procedures using the AxiomTM Analysis Suite (version 5.1.1., Thermo Fisher Scientific, Waltham, MA, USA).

### 2.5. Weightlifting Performance Measurement

Evaluation of the strength of Russian and Japanese weightlifters was assessed by their performance in the snatch and clean and jerk (best results in official competitions including the Olympic Games and the National, European, Asian, and World Championships). The total mass lifted (in kg) was multiplied by the Wilks Coefficient (Coeff) to calculate the standard amount lifted (in point units) normalized across all body mass and sex categories, as previously described [31].

### 2.6. Handgrip Strength Measurement

In addition to the muscle fiber study, 86 individuals (32 females, 54 males) from the initial cohort (*n* = 148) were enrolled in the handgrip strength study. The hand dynamometer (DK-140, St Petersburg, Russia) was used for handgrip strength testing. The strength of both the left and right hands was measured thrice each in a standing position (i.e., with the arm in complete extension and without touching any part of the body with the dynamometer), and the best score of the dominant hand (kg) was used in the analysis.

### 2.7. Search for Genotype–Phenotype Associations Using UK Biobank

A priori, a set of 1535 SNPs was selected to be tested regarding the CSA of fast-twitch muscle fibers in 148 individuals. All of these SNPs showed a genome-wide significance level (*p* < 5 × 10^−8^) in a large previous study involving 450,243 subjects of the UK Biobank cohort [27], as described in summary statistics (https://genetics.opentargets.org/study/GCST90000025 (accessed on 25 May 2024)). Next, we used publicly available summary statistics from genome-wide association studies on handgrip strength, walking pace, and physical activity traits (strenuous sports or other exercises; sports club or gym; vigorous physical activity; number of days/week of vigorous physical activity 10+ min) [32].

### 2.8. Analysis of Association of Muscle Fiber Size-Related SNPs with Gene Expression

The Genotype-Tissue Expression (GTEx) portal [33] was used to analyze the association between muscle fiber size-related SNPs and the expression of genes in human skeletal muscle tissue (m. gastrocnemius; >800 individuals). The GTEx project is an ongoing effort to build a comprehensive public resource to study tissue-specific gene expression and regulation. SNPs that were significantly (*p* < 0.05) correlated with the expression of genes (levels of mRNAs) were considered as expression quantitative trait loci (eQTLs).

### 2.9. Analysis of the Effects of Knockouts of Implicated Genes on Lean Mass and Strength in Mice

Data from the International Mouse Phenotyping Consortium (IMPC) database [34] were used to assess the effects (*p* < 0.05) of gene knockout on absolute and relative lean mass and grip strength in mice.

### 2.10. Analysis of the Effects of Strength Training on the Expression of Muscle Fiber Size-Related Genes

Publicly available human skeletal muscle transcriptome datasets (https://metamex.eu/ (accessed on 25 May 2024)) were used to check the significant effect (*p* < 0.05) of acute (*n* = 198) or chronic (1–52 weeks; *n* = 309) resistance exercise on the mRNA of the muscle fiber size-related genes, as previously described [35].

### 2.11. Statistical Analyses

Haplotype phasing before the imputation of Russian and Japanese microarray data was performed using SHAPEIT2 (v2), and the imputation was performed using IMPUTE2 (v2). For phasing and imputation, we used 1000 Genomes Phase 3 data as a reference panel for Russians and a 3.5 K Japanese reference panel (developed by Tohoku Medical Megabank Organization) for Japanese participants. In a muscle biopsy study, the search for association between 1535 SNPs and the CSA of fast-twitch muscle fibers was performed using logistic regression analysis adjusted for covariates (age, sex, type of training (aerobic, resistance), training frequency, and principal component analysis). Genetic associations for athletic status were analyzed using PLINK v1.9 software under an additive genetic model. For weightlifting performance, linear regression analysis was conducted. All data are presented as mean (standard deviation). *p* values < 0.05 were considered statistically significant.

## 3. Results

### 3.1. Genomic Predictors of Both Appendicular Lean Mass and Muscle Fiber Size

**Figure 1:**
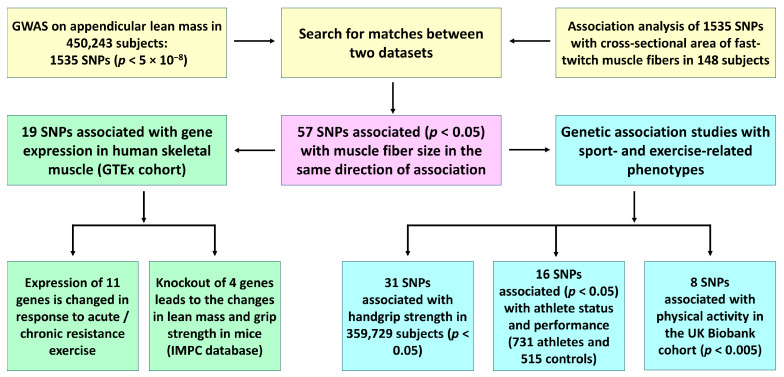
A schematic overview of the study design and the main findings.

A flow diagram displaying the study design and the main findings is shown in Figure 1. We first searched for matches between 1535 SNPs associated with appendicular lean mass at the genome-wide level (*p* < 5 × 10^−8^) and SNPs associated with muscle fiber size (*p* < 0.05) using the CSA of fast-twitch muscle fibers of m. vastus lateralis as a reference. By exploring this approach, we identified a list of 57 independent SNPs (from 57 loci) associated with both appendicular lean mass and the fast-twitch fiber CSA (Table 1). These 57 SNPs showed the same direction of association between the datasets and can be considered as genomic predictors of muscle fiber size.

Given that the fast-twitch fiber CSA was positively correlated with the slow-twitch fiber CSA in our cohort (*r* = 0.64, *p* < 0.0001), we next performed a polygenic analysis with respect to the slow-twitch fiber CSA. Based on 57 SNPs, the number of lean mass-increasing alleles was positively associated with the CSA of slow-twitch muscle fibers of m. vastus lateralis (*p* < 0.0001 adjusted for age, sex, type of training, and training frequency). Furthermore, the positive association between the number of lean mass-increasing alleles and the fast-twitch fiber CSA of m. vastus lateralis remained significant (*p* < 0.0001 adjusted for age, type of training, and training frequency) when we performed a separate analysis for 47 females and 101 males. To determine the functional significance of these SNPs, genetic association studies were performed by exploring phenotypes relevant to sport- and exercise-related traits, as will be shown in the following sections.

### 3.2. Genetic Association Studies with Sport- and Exercise-Related Phenotypes

Given the positive relationship between muscle fiber size and the fiber’s ability to generate muscle force, we checked whether the 57 SNPs associated with appendicular lean mass and the fast-twitch fiber CSA are also associated with handgrip strength in 359,729 subjects from the UK Biobank cohort. A total of 31 of these SNPs also showed an association with handgrip strength (Table 1). Based on these 31 SNPs, the number of lean mass-increasing alleles was positively associated with handgrip strength in 86 participants of the muscle biopsy study (*p* = 0.013 adjusted for age, sex, weight, and type of training).

Furthermore, eight SNPs were associated with physical activity-related phenotypes in the UK Biobank cohort, including walking pace, participation in strenuous sports or other exercises, attending sports club or gym, vigorous physical activity, the number of days/week of vigorous physical activity 10+ min, and the number of days/week walked 10+ min (Table 1). All SNPs have the same direction of association. A complete description of the associations can be found in Supplementary Table S2.

We also conducted case–control association studies using two independent cohorts of athletes (cases) and matched controls from Russia and Japan. Here, the proposal was to verify whether the 57 SNPs associated with appendicular lean mass and the fast-twitch fiber CSA are also associated with athlete status (i.e., whether the SNPs favor the individual to be an athlete) or higher weightlifting performance. A total of sixteen SNPs (twelve in the Russian cohort and four in the Japanese cohort) showed association with at least one athlete status or weightlifting performance, as shown in Table 1. More specifically, six SNPs were associated with elite sprinter status (*E2F7* rs10779153, *VPS52* rs213225, *NADK* rs12040325, *NUDT6* rs12509014, *CENPW* rs853985, and *RAB18* rs2477317); six SNPs were associated with strength (weightlifters and powerlifters) athlete or weightlifter status (*ADAMTS14* rs1420524, *AKAP13* rs11632750, *BOC* rs9810734, *MAPK1* rs34550586, *MERTK* rs55812028, and *PRLR* rs6897259); two SNPs were associated with weightlifting performance (*AKAP13* rs11632750 and *IGFBP3* rs13237404); two SNPs were associated with elite wrestler status (*IGFBP3* rs13237404 and *FERMT1* rs6054078); one SNP was associated with speed–strength athlete status (*MACF1* rs2484749); and one SNP was associated with power athlete status (*LRP5* rs2306862).

In summary, we found that forty-two of the fifty-seven SNPs were associated with appendicular lean mass and the fast-twitch fiber CSA of m. vastus lateralis replicated in at least one association study encompassing a phenotype relevant to exercise and sport. Of these, five SNPs were replicated in two association studies (*HMGA2* rs1480474, *VPS52* rs213225, *PRLR* rs6897259, *ADAMTS14* rs1420524, *MERTK* rs55812028), five SNPs were replicated in three association studies (*LRP5* rs2306862, *CENPW* rs853985, *GIP* rs4794005, *IGFBP3* rs13237404, *NADK* rs12040325), and one SNP (*AKAP13* rs11632750) was replicated in four association studies (handgrip strength + phenotypes related to physical activity + athlete status + weightlifting performance).

### 3.3. Bioinformatic Analyses Using Publicly Available Data

Based on eQTL data available on the GTEx portal, 19 of the 57 SNPs (associated with appendicular lean mass and the fast-twitch fiber CSA) correlate with changes in gene expression in human skeletal muscle (m. gastrocnemius), as shown in Table 2. In line with these eQTL data, we checked whether the genes to which these SNPs belong were significantly regulated in skeletal muscle by acute/chronic resistance exercise (using a previously published database) [35]. We found that of the nineteen genes, four genes were regulated by the resistance exercise session (acute effect), five genes were regulated by resistance exercise training (chronic effect), and two genes were regulated by both the acute and chronic effects (Table 3). Furthermore, it was found in an animal model that the knockout of four of these genes (*Camkmt*, *Igfbp3*, *Nudt6*, and *Pabpc4*) significantly influences lean mass or grip strength (Supplementary Table S2). These influences have been reported in the same direction as genetic association.

## 4. Discussion

In the present study, we explored whether 1535 genetic variants previously reported to be associated with appendicular lean mass at the genome-wide level [27] are also associated with the CSA of fast-twitch muscle fibers of m. vastus lateralis in physically active individuals. Pei et al. [27] was the largest sample used for a GWAS of lean mass so far, with approximately half a million participants, and the identified SNPs accounted for a significant fraction of the appendicular lean mass variation (phenotypic variance)—a complex trait with high heritability. The large sample size assessed in this GWAS enhanced the statistical power to detect causal SNPs underlying lean body mass. Our proposal was to evaluate whether these SNPs strongly associated with lean mass would also be associated with a direct measurement of the CSA of fast-twitch muscle fibers since these muscle fibers are more responsive to muscle hypertrophy and force generation.

After searching for matches between the lean mass [27] and fast-twitch muscle fiber CSA datasets, we identified and explored 57 SNPs associated with both appendicular lean mass and the CSA of fast-twitch muscle fibers. Of the 57 SNPs, 31 were also associated with handgrip strength in the UK Biobank cohort. Importantly, we were able to replicate this association in our muscle biopsy study using a polygenic approach and handgrip strength data. These findings reinforce a shared biology between muscle mass (fiber size) and function (muscle strength).

This may have consequences for sports performance but also for different health-related phenotypes. Muscle morphology and plasticity are heritable traits associated with disease prevention (preservation of physical and metabolic health) and sports performance (strength and power performance). Greater lean mass is a protective factor against metabolic diseases, such as type 2 diabetes and obesity-related traits [27]. Furthermore, muscle fiber size is also an important trait in the field of sarcopenia/muscle aging research [36]. It has previously been shown that two SNPs (*MLN* rs12055409 and *GBF1* rs2273555) associated with higher levels of muscle mass and strength in elite Russian weightlifters [22] protect against sarcopenia [28]. Therefore, the translational value of these findings lies in the importance of lean mass for muscle-related traits and complex diseases.

Based on the premise that greater muscle fiber size improves muscle performance on a daily basis, it was verified whether the 57 SNPs associated with appendicular lean mass and the CSA of fast-twitch muscle fibers are also associated with sport- and exercise-related phenotypes in data from the UK Biobank. It was found that 33 SNPs were associated with phenotypes such as participation in strenuous sports or other exercises, attendance at sports clubs or gyms, usual walking pace, vigorous physical activity, and others. Furthermore, favorable alleles/genotypes of 16 SNPs were overrepresented in elite or highly elite power athletes from Russia or Japan. Taken together, these data reinforce the partial influence of these SNPs on muscle-related traits and athlete status. It is a partial influence because there are certainly other undetected polymorphisms and numerous environmental factors influencing these complex phenotypes. The predisposition to success in sports is related to the number of favorable alleles the individual has [37] and their interaction with appropriate environmental factors. Even a “more isolated” phenotype, such as the CSA of fast-twitch muscle fibers, has a complex and polygenic nature [22,24].

The identified 57 SNPs are located in or near 57 different genes that have multiple functions, including cell division and growth, transcriptional or translational regulation, cell renewal, and energy metabolism. Given the pleiotropic effect, it is important to note that these genes may have a function not yet known in skeletal muscle or related tissues. Interestingly, of the fifty-seven SNPs, nineteen were identified as eQTL SNPs that correlated with gene expression in human skeletal muscle, and eight of these genes were shown to be influenced by exercise or resistance training [35] in the same direction as the genetic association. Given that resistance exercise stimulation is one of the most common and efficient ways to induce muscular hypertrophy, it is plausible to assume that carriers of favorable alleles in these SNPs are more responsive to the increase in the CSA of fast-twitch muscle fibers. Furthermore, of the nineteen eQTL SNPs, the knockouts of four genes significantly affect lean mass and grip strength in mice in the same direction as the genetic association, reinforcing the functional relevance of our identified SNPs in muscle-related traits.

Overall, we found 57 new genomic predictors of muscle fiber size, which significantly exceeds the number of known genetic markers of muscle fiber hypertrophy (10 alleles with hypertrophic effects: *AGRN* rs4074992 C, *DOCK3* rs77031559 G, *ESR1* rs190930099 G, *GLIS3* rs34706136 TG, *GRAMD1B* rs850294 T, *MLN* rs12055409 G, *PPARG* rs1801282 G, *TRAIP* rs62260729 C, *UBR5* rs10505025 A, *UBR5* rs4734621 A) [20,22,23,24,25]. Furthermore, our results expand the existing list of genetic markers associated with strength athlete, sprinter, and power athlete statuses, as well as weightlifting performance [31,38,39].

Our study does have limitations. Firstly, genome-wide SNPs were identified in individuals of European ancestry from across the United Kingdom, and populations of other origins or admixtures may not be covered by these SNPs. Second, although we included 731 athletes and 515 controls in our case–control association studies, when stratified by subgroups, comparisons were made between groups with limited sample sizes. Third, only athletes of Russian or Japanese origin were analyzed, and we strongly recommend that other athlete populations be evaluated to test the relevance of these genetic markers, especially larger and homogenous cohorts. In the future, the addition of new independent samples of athletes will enable us to conduct a meta-analysis and establish the significance of each marker [40]. Some SNPs may not have been replicated in the present study due to the sample size of top-level athletes. We also encourage the exploratory study of the genes and SNPs described here in other physiological and molecular contexts, such as the skeletal muscle hypertrophy response to resistance training [41]. Fourth, we acknowledge that there are many molecular differences between human and mouse skeletal muscle. Therefore, our findings regarding mouse knockout models should be interpreted with caution. Finally, instead of performing a GWAS of the CSA of fast-twitch muscle fibers, we used a candidate gene approach with already discovered genetic variants reported to be associated with appendicular lean mass in a large cohort of individuals. This was performed intentionally since conducting GWASs with relatively small sample sizes is unproductive and carries the risk of identifying many false-positive associations. In contrast, the use of genetic variants that have previously been shown to be associated with closely related phenotypes, such as lean mass and muscle fiber size, at the genome-wide significance level (*p* < 5 × 10^−8^) leads to the discovery of a large number of significant genetic markers [42].

## 5. Conclusions

In conclusion, we identified 57 genetic variants associated with both appendicular lean mass and fast-twitch muscle fiber size of m. vastus lateralis that may, in part, contribute to a greater pre-disposition to power sports. Given that we are evaluating a complex phenotype, we suspect that the 57 polymorphisms we have identified constitute only a small fraction of the genetic factors that influence muscle fiber size. However, at present, this represents a set of polymorphisms with potential relevance for muscle-related traits and athletic performance. It would be interesting to carry out replication studies on independent cohorts to further investigate these findings.

## Figures and Tables

**Table 1:** Single-nucleotide polymorphisms associated with appendicular lean mass, fast-twitch fiber size, and exercise- and sport-related phenotypes.

Nearest Gene	SNP	Allele 1/Allele 2	Favorable Allele	*p* Value (app. Lean Mass)	*p* Value (CSA of Fast-Twitch Muscle Fibers)	*p* Value (Phenotype)
*AKAP13*	rs11632750	A/C	C	3.9 × 10^−37^	0.032	0.012 (HS); 0.048 (WL); 0.035 (WP); 0.00016 (PA)
*LRP5*	rs2306862	C/T	C	1.4 × 10^−46^	0.041	3.4 × 10^−9^ (HS); 0.03 (POW); 0.0007 (PA)
*CENPW*	rs853985	C/T	C	4.7 × 10^−121^	0.017	0.000016 (HS); 0.014 (SPR); 0.0027 (WAL)
*GIP*	rs4794005	G/A	A	5.3 × 10^−22^	0.025	0.00043 (HS); 0.000094 (PA); 0.0011 (WAL)
*IGFBP3*	rs13237404	G/A	G	5.6 × 10^−27^	0.045	0.0001 (HS); 0.006 (WP); 0.03 (WRS)
*NADK*	rs12040325	G/A	A	1.0 × 10^−10^	0.027	0.0013 (HS); 0.011 (SPR); 0.0035 (PA)
*HMGA2*	rs1480474	A/G	A	1.1 × 10^−139^	0.023	1.5 × 10^−12^ (HS); 0.0042 (PA)
*VPS52*	rs213225	G/A	A	6.8 × 10^−9^	0.00008	0.038 (SPR); 0.00068 (PA)
*PRLR*	rs6897259	T/C	C	9.6 × 10^−14^	0.018	0.00018 (HS); 0.03 (STR)
*ADAMTS14*	rs1420524	T/A	A	1.0 × 10^−12^	0.042	0.007 (WL); 0.023 (STR)
*MERTK*	rs55812028	C/T	T	3.7 × 10^−13^	0.019	0.016 (WL); 0.049 (STR)
*L3MBTL3*	rs7740107	T/A	T	7.5 × 10^−99^	0.046	6.3 × 10^−21^ (HS)
*PHF20*	rs6121042	C/T	C	1.3 × 10^−114^	0.048	3.5 × 10^−13^ (HS)
*TSBP1*	rs9268249	T/A	T	9.1 × 10^−42^	0.016	6.0 × 10^−9^ (HS)
*CEP120*	rs34732995	C/CTA	C	2.2 × 10^−55^	0.017	4.6 × 10^−8^ (HS)
*FIS1*	rs4729677	A/G	A	1.2 × 10^−10^	0.042	9.1 × 10^−7^ (HS)
*MECOM*	rs2115959	A/C	C	5.7 × 10^−14^	0.038	0.0000015 (HS)
*DCST1*	rs150352963	C/G	G	1.3 × 10^−17^	0.042	0.000025 (HS)
*IGF1*	rs35762	T/C	T	8.4 × 10^−21^	0.002	0.000055 (HS)
*CAMKMT*	rs343954	T/C	C	5.4 × 10^−10^	0.044	0.000062 (HS)
*ZFAT*	rs137957419	I/D	D	1.7 × 10^−18^	0.024	0.00012 (HS)
*MLST8*	rs26866	A/G	G	2.9 × 10^−28^	0.006	0.00022 (HS)
*NUDT6*	rs12509014	C/T	C	5.4 × 10^−30^	0.038	0.0007 (SPR)
*FOXD2*	rs10749868	C/T	C	4.8 × 10^−9^	0.005	0.00085 (HS)
*NDUFS4*	rs7727774	A/G	A	7.5 × 10^−13^	0.013	0.00094 (HS)
*ATAD2B*	rs4665244	A/G	G	1.5 × 10^−31^	0.027	0.001 (HS)
*NTAN1*	rs3803573	C/T	C	1.8 × 10^−15^	0.019	0.0011 (HS)
*TREH*	rs472419	C/T	T	8.8 × 10^−11^	0.012	0.0013 (PA)
*NOTCH4*	rs8192589	G/T	T	5.4 × 10^−51^	0.010	0.0023 (HS)
*C4A*	rs693906	G/C	C	2.2 × 10^−54^	0.015	0.0029 (HS)
*BTNL2*	rs2227138	C/T	T	7.0 × 10^−44^	0.026	0.0036 (HS)
*PMAIP1*	rs8086627	C/A	A	2.0 × 10^−45^	0.017	0.005 (HS)
*HLA-DQA1*	rs9271657	T/C	C	4.1 × 10^−34^	0.046	0.006 (HS)
*RAB18*	rs2477317	A/G	G	3.3 × 10^−11^	0.034	0.0065 (SPR)
*FERMT1*	rs6054078	A/C	C	1.6 × 10^−9^	0.019	0.01 (WRS)
*ADAMTS6*	rs9291834	T/C	T	2.8 × 10^−8^	0.019	0.012 (HS)
*BOC*	rs9810734	A/T	T	2.3 × 10^−8^	0.035	0.02 (STR)
*E2F7*	rs10779153	T/A	A	1.5 × 10^−9^	0.00007	0.024 (SPR)
*PITX2*	rs2595104	T/G	T	1.2 × 10^−11^	0.048	0.033 (HS)
*MACF1*	rs2484749	A/G	A	4.9 × 10^−10^	0.046	0.038 (SPS)
*MAPK1*	rs34550586	A/G	G	1.5 × 10^−9^	0.021	0.041 (WL)
*RUNX2*	rs1321080	G/T	G	2.0 × 10^−16^	0.021	0.049 (HS)
*CASZ1*	rs11121615	C/T	C	3.3 × 10^−23^	0.036	NS
*PABPC4*	rs3768320	T/C	T	3.0 × 10^−10^	0.034	NS
*PLPP3*	rs12140284	T/C	C	3.3 × 10^−33^	0.028	NS
*CIART*	rs2318761	A/G	A	8.2 × 10^−17^	0.020	NS
*RPS6KC1*	rs182673203	C/T	T	6.2 × 10^−11^	0.029	NS
*DIS3L2*	rs79057767	A/G	G	3.2 × 10^−21^	0.004	NS
*CMSS1*	rs35225638	T/TG	TG	4.8 × 10^−12^	0.045	NS
*RBPJ*	rs3109841	G/T	T	5.9 × 10^−14^	0.026	NS
*ADAMTS3*	rs72852033	T/C	T	2.4 × 10^−10^	0.006	NS
*WDR70*	rs4869505	G/C	C	6.5 × 10^−31^	0.032	NS
*VTI1A*	rs11196067	A/T	T	2.1 × 10^−9^	0.004	NS
*STXBP6*	rs61981417	C/T	C	2.3 × 10^−8^	0.037	NS
*PAQR5*	rs2415040	G/C	G	1.0 × 10^−8^	0.028	NS
*TLE3*	rs2291982	C/A	A	6.4 × 10^−9^	0.039	NS
*NTN1*	rs7223668	A/G	G	2.2 × 10^−11^	0.049	NS

HS, handgrip strength; SPR, sprinter status; SPS, speed–strength athlete status; WL, weightlifter status; WP, weightlifting performance; STR, strength athlete status; POW, power athlete status; WRS, wrestler status; PA, physical activity; WAL, walking pace; SNP, single-nucleotide polymorphism; CSA, cross-sectional area; NS, not significant.

**Table 2:** Association between muscle fiber size-related SNPs and the expression of affected genes in skeletal muscle of the GTEx cohort.

Gene/Near Gene	SNP	Favorable Allele	Effect of Favorable Allele on Gene Expression
*NADK*	rs12040325	A	*NADK* ↑ (β = 0.11; *p* = 1.3 × 10^−8^)
*MACF1*	rs2484749	A	*MACF1* ↑ (β = 0.13; *p* = 0.00011)
*PABPC4*	rs3768320	T	*PABPC4* ↓ (β = −0.12; *p* = 0.0000013)
*CIART*	rs2318761	A	*CIART* ↓ (β = −0.068; *p* = 0.05)
*ATAD2B*	rs4665244	G	*ATAD2B* ↑ (β = 0.045; *p* = 0.05)
*CAMKMT*	rs343954	C	*CAMKMT* ↑ (β = 0.14; *p* = 0.0011)
*NUDT6*	rs12509014	C	*NUDT6* ↑ (β = 0.20; *p* = 1.5 × 10^−13^)
*NDUFS4*	rs7727774	A	*NDUFS4* ↓ (β = −0.043; *p* = 0.0091)
*CEP120*	rs34732995	C	*CEP120* ↑ (β = 0.075; *p* = 0.0011)
*C4A*	rs693906	C	*C4A* ↓ (β = −0.57; *p* = 8.2 × 10^−22^)
*NOTCH4*	rs8192589	T	*NOTCH4* ↓ (β = −0.34; *p* = 5.2 × 10^−11^)
*L3MBTL3*	rs7740107	T	*L3MBTL3* ↑ (β = 0.25; *p* = 6.5 × 10^−10^)
*IGFBP3*	rs13237404	G	*IGFBP3* ↓ (β = −0.14; *p* = 0.0054)
*AKAP13*	rs11632750	C	*AKAP13* ↓ (β = −0.051; *p* = 0.00027)
*MLST8*	rs26866	G	*MLST8* ↑ (β = 0.24; *p* = 4.6 × 10^−21^)
*NTAN1*	rs3803573	C	*NTAN1* ↑ (β = 0.072; *p* = 0.0031)
*FERMT1*	rs6054078	C	*FERMT1* ↓ (β = −0.095; *p* = 0.024)
*PHF20*	rs6121042	C	*PHF20* ↑ (β = 0.045; *p* = 0.037)
*MAPK1*	rs34550586	G	*MAPK1* ↓ (β = −0.15; *p* = 5.0 × 10^−10^)

↑, increased gene expression; ↓ decreased gene expression. β, normalized effect size.

**Table 3:** Effects of acute resistance exercise and resistance training on the expression of muscle fiber size-related genes.

Gene/Near Gene	Effect of RE on Gene Expression	Effect of RT on Gene Expression
*NADK*	*NADK* ↑	NS
*MACF1*	*MACF1* ↑	*MACF1* ↑
*PABPC4*	NS	*PABPC4* ↓
*CIART*	*CIART* ↓	*CIART* ↓
*CAMKMT*	*CAMKMT* ↑	NS
*NUDT6*	NS	*NUDT6* ↑
*NDUFS4*	NS	*NDUFS4* ↓
*NOTCH4*	*NOTCH4* ↓	NS
*AKAP13*	NS	*AKAP13* ↓
*NTAN1*	NS	*NTAN1* ↑
*PHF20*	*PHF20* ↑	NS

↑, increased gene expression; ↓ decreased gene expression. NS, not significant. All gene expressions were evaluated in human skeletal muscle. RE, resistance exercise session (acute effect). RT, resistance training (chronic effect). A complete description of the associations can be found in Supplementary Table S2.

## Data Availability

The human data presented in this study are publicly available online at https://genetics.opentargets.org (accessed on 25 May 2024) and https://gtexportal.org/home/index.html (accessed on 25 May 2024). The mouse knockout data are publicly available online at https://www.mousephenotype.org (accessed on 25 May 2024).

## Supplementary Materials

The following supporting information can be downloaded at https://www.mdpi.com/article/10.3390/cells13141212/s1, Table S1: Characteristics of 148 physically active participants; Table S2: Association of lean mass-related SNPs with muscle fiber size and other phenotypes; Figure S1: Microphotographs of the labeled muscle sections (immunohistochemistry).
